# CAR-T and BiTE: new horizons in the treatment of rheumatic autoimmune diseases

**DOI:** 10.3389/fimmu.2026.1747777

**Published:** 2026-01-30

**Authors:** Jie Li, Qianyu Guo, Linxin Li, Juanjuan Wang, Liyun Zhang

**Affiliations:** 1 Third Hospital of Shanxi Medical University, Shanxi Bethune Hospital, Shanxi Academy of Medical Sciences, Tongji Shanxi Hospital, Taiyuan, China; 2 Department of Rheumatology and Immunology, Shanxi Bethune Hospital, Shanxi Academy of Medical Sciences, Third Hospital of Shanxi Medical University, Tongji Shanxi Hospital, Taiyuan, China

**Keywords:** bispecific T cell engager, CAR-T, clinical efficacy, immunotherapy, rheumatic immune diseases

## Abstract

Autoimmune diseases arise from immune system dysfunction, in which immune cells erroneously attack the body’s own tissues, leading to systemic disorders or localized pathological changes. The number of patients with autoimmune diseases is gradually increasing, and patients with relapsing-refractory conditions face the dilemma of inadequate efficacy when treated with conventional medications and biologic agents. However, bispecific T-cell engagers (BiTEs) and chimeric antigen receptor T-cell (CAR-T) therapy, as emerging immunotherapeutic strategies, have opened up new possibilities for the treatment of these diseases. BiTEs activate T-cell-mediated immune responses by simultaneously targeting T cells and tumor-associated antigens, while CAR-T therapy involves genetic engineering of T cells to enable them to specifically recognize and eliminate target cells. Both therapeutic approaches have demonstrated unique advantages and potential in the treatment of rheumatic immune diseases, providing novel insights and methods to address this challenging clinical issue. This article will conduct a comparative analysis of the applications of CAR-T cell therapy and BiTEs in rheumatic immune diseases, exploring their mechanisms of action, therapeutic efficacy, safety profiles, and future development prospects, with the aim of providing references for clinical practice.

## 1 Introduction

Autoimmune diseases represent a category of disorders defined by dysregulation of the immune system, wherein the cardinal pathological feature arises from a defective capacity to distinguish self from non-self, thereby inducing aberrant immune-mediated assaults against the host’s own cellular and tissue components (1, 2). In recent years, the population of autoimmune diseases has been increasing gradually and showing a trend of youth, in general, these patients are required to undergo long-term or lifelong pharmacotherapy for disease control. which brings more pain and economic burden; for patients with relapsed and refractory diseases that have not been improved by traditional medications, the emerging monoclonal antibody-based biologics have not been effective in some of them (3), Chimeric antigen receptor T-cell (CAR-T cell) therapy and bispecific antibody drugs have demonstrated remarkable efficacy in the field of oncology treatment. Based on their therapeutic mechanisms, these approaches are now also being explored for application in autoimmune diseases.

Accumulating evidence has identified B lymphocytes as critical contributors to the pathogenic mechanisms underlying autoimmune disorders, including rheumatoid arthritis and systemic lupus erythematosus. It coordinates antigen presentation, produces cytokines, and some of them can further differentiate into antibody-secreting plasma cells, which are involved in disease development (4–6).

T lymphocytes play a pivotal role in preserving immune cell equilibrium and host protective mechanisms, while concurrently serving as primary drivers of pathogenic processes in autoimmune and inflammatory disorders. Based on their cellular profiles, T cells are divided into subpopulations, each of which secretes different cytokines involved in pro- or anti-inflammatory processes. Abnormalities in T-cell immunity can trigger or contribute to the development of autoimmune diseases (7, 8).

The interplay between B lymphocytes and T lymphocytes (B-T cell crosstalk) has been implicated in the pathogenesis of autoimmune conditions, including rheumatoid arthritis and systemic lupus erythematosus (9–11). This synergy can occur through cytokines or other contact immune stimuli. This interaction exists at the site of inflammation in a variety of autoimmune diseases. Rao et al. identified a class of PD1+CXCR3+CXCR5-T cells in synovial tissues of patients with RA, which can stimulate B-cell maturation by secreting IL-10 (9–11).

Both chimeric antigen receptor T-cell (CAR-T cell) therapy and bispecific T-cell engagers (BiTEs) harness T-cell cytotoxicity to disrupt this B-T cell crosstalk. Currently, these two novel therapeutic modalities are being explored for the treatment of autoimmune diseases; however, their comparative safety profiles, therapeutic efficacy, and optimal patient populations remain unclear. In this study, we elaborate on and compare these aspects based on the currently available data.

## 2 Mechanism of action and production process

### 2.1 Bispecific T cell engager production process and mechanism of action

**Figure 1:**
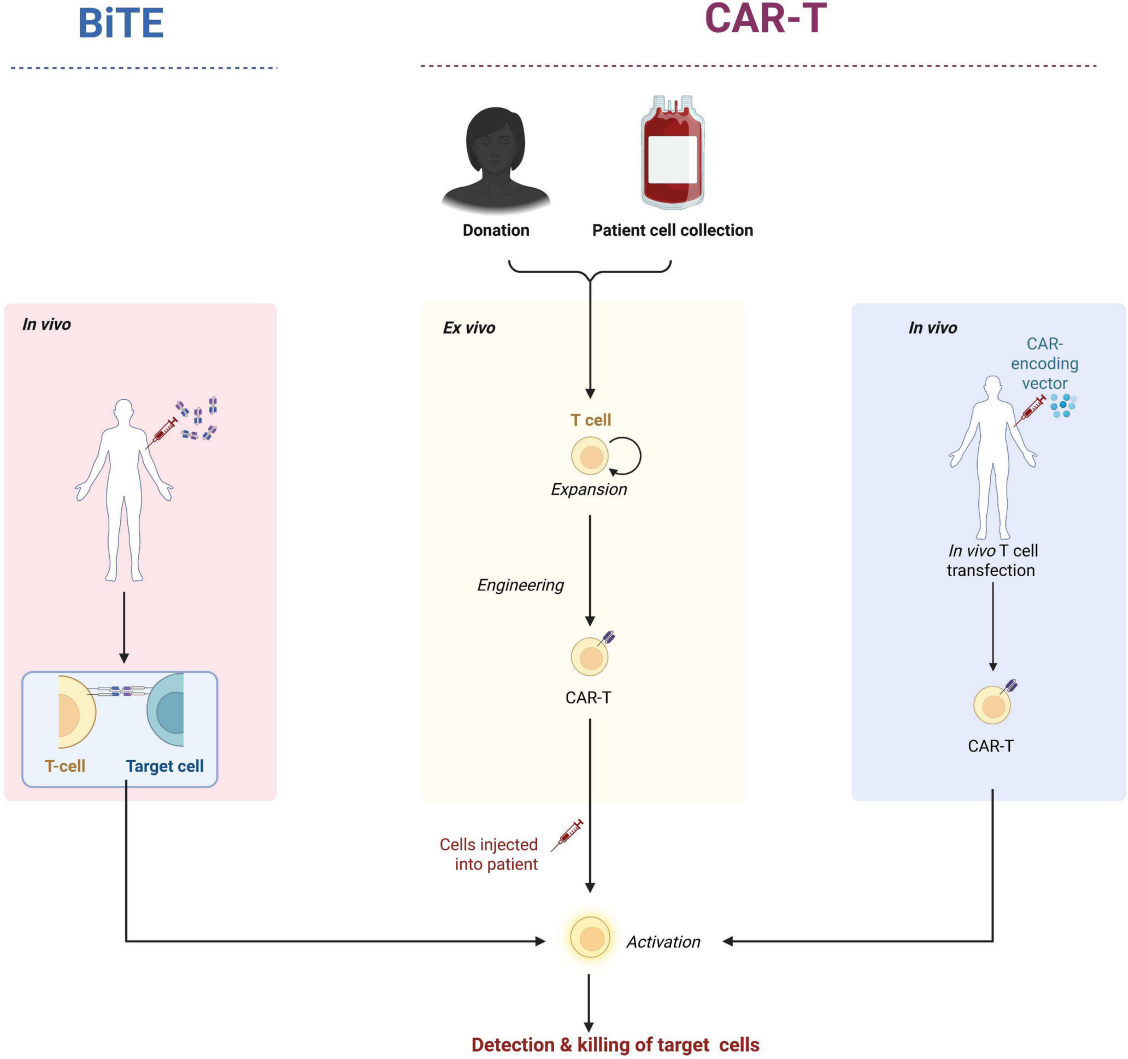
Manufacturing processes and mechanisms of action of CAR-T and bispecific T cell engagers.

Structurally, bispecific T-cell engagers (BiTEs) consist of two single-chain variable fragments (scFvs). One of the scFvs specifically binds to the CD3 molecule on the T-cell surface—a key signaling molecule for T-cell activation—while the other scFv specifically targets a distinct antigen on the surface of target cells, such as tumor-associated antigens or pathogenic antigens in autoimmune diseases. Through this bispecific binding, BiTEs act as a “bridge” to tightly connect T cells and target cells, narrowing the distance between them and creating conditions for subsequent immune-mediated cytotoxic responses (12–14). Upon simultaneous binding of BiTE molecules to T cells and target cells, a cascade of immune responses is triggered. Activation of the CD3 molecule on the T-cell surface represents a critical step in T-cell activation. Activated T cells then release a variety of cytotoxic substances, such as perforin and granzyme. Perforin is capable of forming pores in the target cell membrane, enabling substances like granzyme to enter the interior of target cells. Once inside, granzyme activates a series of apoptotic signaling pathways, ultimately inducing target cell apoptosis and subsequent clearance (15). This mechanism of action enables BiTEs to precisely direct T cells to eliminate target cells, independent of antigen-presenting cells (APCs) and the antigen-presenting function of major histocompatibility complexes (MHCs). However, BiTEs have limitations, including a short half-life that necessitates multiple infusions. In contrast, the manufacturing process of BiTEs is relatively straightforward, with high operability and lower costs compared to CAR-T cell therapy. The production workflow involves first generating two distinct monoclonal antibodies separately. Subsequently, appropriate amounts of these two antibodies are mixed with a cross-linking agent under specific conditions to induce cross-linking between antibody molecules. Following the completion of the cross-linking reaction, purification is performed to obtain the bispecific antibody product (**Figure 1**). Currently, this therapeutic modality is also being explored for the treatment of rheumatic autoimmune diseases (16, 17).

### 2.2 CAR-T production process and mechanism of action

Chimeric antigen receptor T-cell (CAR-T) therapy is an emerging adoptive T-cell therapy. The manufacturing process of *ex vivo* CAR-T cells involves multiple critical steps. First, T cells are collected from the patient’s body. As a crucial component of the immune system, T cells possess the ability to recognize and attack pathogens and abnormal cells. Through isolation techniques, T cells are separated from the patient’s blood and transported to specialized laboratories, where they undergo genetic engineering modification (**Figure 1**). The objective of this modification is to introduce the chimeric antigen receptor (CAR) gene into T cells (18–20). Functioning like a “navigation system” installed in T cells, CAR enables specific recognition and binding to distinct antigens on the surface of tumor cells or pathogenic cells, thereby endowing T cells with the capacity for precise targeting and attack. Unlike BiTEs, CAR-T cells can persist and proliferate long-term *in vivo*. Starting from the second-generation CAR-T cells, these cells have been engineered to exhibit an effector memory phenotype and possess sustained activation properties. However, the manufacturing process of CAR-T cells is relatively complex. Autologous CAR-T cells require personalized customization, which results in prolonged production time and high costs—barriers that deter many patients from accessing this therapy (21). To address the limitations of *ex vivo* CAR-T therapy, *in vivo* CAR-T therapy has been developed as a promising alternative. Its core advantage lies in the ability to achieve direct delivery and expression of CAR genes within the patient’s body, eliminating the need for *ex vivo* isolation, genetic modification, and expansion of autologous T cells. CAR gene delivery is primarily mediated by two categories of vectors: viral vectors (e.g., adeno-associated virus [AAV], lentivirus [LV]) and non-viral vectors (e.g., lipid nanoparticles [LNPs], targeted nanocarriers). The injected CAR gene-vector complexes recognize and bind to specific surface receptors on T cells (e.g., CD3, CD4) via vector-conjugated targeting moieties. Subsequently, the CAR genes are internalized into T cells through vector-specific delivery mechanisms, such as endocytosis for AAV and membrane fusion for LNPs. Once inside T cells, CAR genes undergo transcription and translation, and the synthesized CAR molecules are anchored to the T cell membrane via their transmembrane domains. This *in situ* T cell reprogramming equips naive T cells with the capacity to specifically recognize and target SLE-associated pathogenic cells. Despite these advances, *in vivo* CAR-T therapy still faces critical challenges, including suboptimal vector delivery efficiency, insufficient targeting specificity, short persistence, and unresolved long-term safety concerns, all of which necessitate further optimization for clinical translation (22).

## 3 Application of CAR-T cells and bispecific T-cell engagers in rheumatic autoimmune diseases

### 3.1 Application of CAR-T cells in the treatment of rheumatic autoimmune diseases

**Figure 2:**
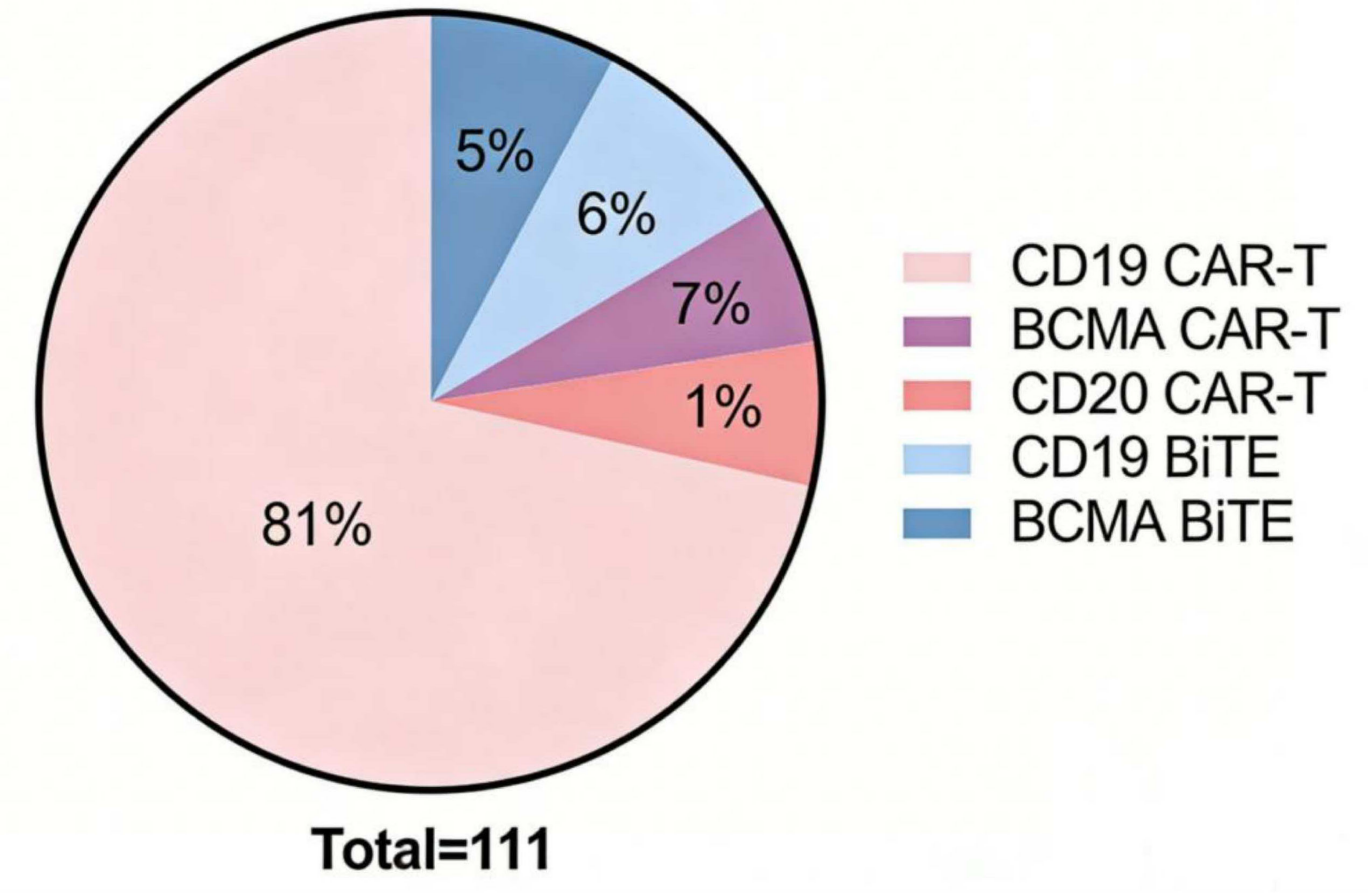
Proportions of cases treated with CAR - T or Bispecific T - cell engager drugs in the reported cases of rheumatic immune diseases, as well as the proportions of CD19, CD20, and BCMA targets (As of December 1, 2025).

Chimeric antigen receptor T-cell (CAR-T cell) therapy has shown promising prospects in the field of oncology. Based on its therapeutic mechanism, this therapy has also begun to be explored for application in autoimmune diseases. Next, we will discuss the clinical progress of CAR-T cell therapy in the treatment of rheumatic autoimmune diseases. To date, rheumatic autoimmune diseases for which CAR-T cell clinical trials have been initiated include systemic lupus erythematosus, Sjögren’s syndrome, systemic sclerosis, inflammatory myopathies, and rheumatoid arthritis (23) (**Table 1**), with variations in the selection of therapeutic targets (**Figure 2**).

**Table 1:** Published case reports on the use of CAR-T therapy in rheumatic immune diseases to date (As of December 1, 2025).

Target	autologous/Allogeneic	Location	Disease	Number of patients	Average age(Age range)	Male /Female	Average disease duration(Disease duration range)	Disease remission rate	CRS(≥ grade 3)	Neurotoxicity	Hematotoxicity	Chronic dysplasia of B cells	Hypogammaglobulinemia: Present/Absent	Infection: Present/Absent	Medication use after treatment (during follow-up)
CD19	autologous	Germany	SSc	1	38	0/1	4m	100%	–	–	–	NA	NA	1/0	Maintenance with nintedanib and mycophenolate mofetil
CD19	Allogeneic	China	SSc,IIM	3(2SSc,1IIM)	48(42~56)	2/1	4.7(1~10)y	100%	–	–	–	–	0/3	0/3	Medication-free remission
CD19	autologous	Germany	SSc	1	60	1/0	28m	100%	–	–	–	–	0/1	0/1	Medication-free remission
CD19	autologous	Germany	SLE,IIM,SSc	15(8SLE,3IIM,4SSc)	35(18~60)	6/9	5.7(1~20)y	100%	–	One case of mild ICANS	+(One case developed neutropenia)	–	5/10	8/7	Medication-free remission
CD19	autologous	USA	SLE	5	22(18~24)	1/4	4(1~9)y	100%	–	–	–	–	0/5	0/5	Medication-free remission
CD19	autologous	China	SLE	3	36(18~54)	0/3	13(9~19)y	100%	–	–	+(severe thrombocytopenia.	–	0/5	1/2	Medication-free remission
CD19/BCMA	Allogeneic	China	SLE	15	30(19~43)	1//14	–	100%	–	–	–	–	0/15	9/6	Medication-free remission
CD19	autologous	China	SLE	2	12	0/2	4(3~5)y	100%	–	One case of mild ICANS	–	+	0/2	0/2	Medication-free remission
CD19	autologous	China	SLE	8	28(21~36)	0/8	9(2~21)y	100%	+(Two patients experienced grade 3 CRS)	–	+	–	5/3	1/8	Medication-free remission
CD19	autologous	USA	SLE	1	30	0/1	8y	100%	–	–	+(Leukopenia)	+	1/0	1/0	Drug-free remission was achieved at 6 months following CAR-T therapy.
CD19	autologous	China	SLE	8	31(20~36)	0/8	10(2~19)y	62.50%	–	One case of mild ICANS	–	–	0/1	NA	Five cases achieved drug-free remission
CD19	autologous	Germany	IIM	1	54	0/1	3y	100%	–	–	+(Hypohemoglobinemia)	–	0/1	1/0	Medication-free remission
CD19	autologous	Germany	RA,SSc	1	32	0/1	6y	100%	–	–	+(One case developed neutropenia)	–	0/1	1/0	Low-dose prednisone (2 mg/day) was continued until week 6 after CAR-T therapy
CD19	autologous	Germany	SSc	6	42(36–53)	4/2	4(1~11)y	100%	–	–	+	–	0/1	6/0	Three of the patients continued to use vasodilators
CD19	autologous	Germany	IIM	1	41	1/0	26m	100%	–	–	–	–	0/1	NA	Mycophenolate mofetil was continued 35 days after CAR-T infusion
CD19	autologous	Germany	IIM	1	41	1/0	18m	100%	–	–	–	–	0/1	0/1	Medication-free remission
CD19	autologous	Italy	IIM	1	12	1/0	6y	100%	–	–	+(Transient anemia(Grade 2)and neutropenia(Grade 4))	–	0/1	0/1	Medication-free remission
CD19	autologous	Germany	IIM	1	44	0/1	6y	100%	–	Grade 1 ICANS	NA	–	NA	NA	Medication-free remission
CD19	autologous	USA	IIM	1	33	1/0	23m	100%	–	–	Peripheral leukopenia	–	NA	NA	Medication-free remission
BCMA	autologous	China	IIM, SS	1	25	1/0	7y	100%	–	–	–	–	1/0	NA	Medication-free remission
CD19	autologous	Spain	IIM	1	10	1/0	4m	100%	–	–	NA	+	1/0	1/0	Medication-free remission
CD19	autologous	Germany	SS	1	76	0/1	10y	100%	–	Grade 1	+(Grade 3 anemia and Grade 4 thrombocytopenia)	–	1/0	1/0	Medication-free remission
CD19	autologous	China	RA	3	52(49~56)	0/3	–	100%	–	–	+(One case developed neutropenia)	–	NA	1/2	Patient 1: Half-dose of hormones + hydroxychloroquine + non-steroidal drugs (as needed);Patient 2: Half-dose of hormones;Patient 3: Hydroxychloroquine
CD19	autologous	Germany	RA, Myasthenia gravis	1	37	0/1	11y	100%	–	–	–	–	0/1	0/1	Prednisolone 4 mg/day and acetylcholinesterase inhibitors are used to prevent rebound of disease activity and will be gradually reduced
CD20-CD19	autologous	Germany	RA, DLBCL	1	73	1/0	13y	100%	–	–	–	–	0/1	NA	Medication-free remission

Systemic lupus erythematosus (SLE) is a systemic autoimmune disease involving multiple organs and systems (24, 25). While disease activity can be effectively controlled with standardized treatment in most patients, a subset experiences disease relapse or vital organ involvement, leading to severe complications that impair quality of life and reduce survival. Thus, there is an urgent need for novel therapeutic strategies. B cells play a critical role in SLE pathogenesis, and most emerging therapies target B cell depletion for therapeutic efficacy (26). CD19 is widely expressed on B lymphocytes, and CD19-targeted CAR-T cells can specifically eliminate B cells. Significant progress has been made in the application of CAR-T therapy for SLE, with CD19 as the most commonly used target. In August 2021, the Georgschett team reported the first case of SLE treated with CAR-T therapy (27). Subsequent studies enrolled additional SLE patients, and long-term follow-up showed that 100% of patients achieved complete short-term symptom remission and complete glucocorticoid independence. To date, 66 cases of SLE treated with CAR-T therapy have been reported, including 9 from Germany (28), 48 from China (29–34), and 9 from the United States (35, 36). Favorable efficacy and safety profiles were observed, with no high-grade adverse events reported.

Sjögren’s syndrome (SS) is a common autoimmune disease primarily affecting exocrine glands (especially salivary and lacrimal glands), leading to xerostomia and xerophthalmia (37). In SS, excessive B cell activation results in the production of autoantibodies (e.g., anti-SS-A and anti-SS-B antibodies), triggering chronic inflammation of exocrine glands (38). B cell-targeted CAR-T therapy induces profound B cell depletion and immune reconstitution in autoimmune disease patients, thereby exerting therapeutic effects. To date, only 2 clinical reports on CAR-T therapy for SS have been published. Both patients achieved drug-free remission following CD19-targeted CAR-T therapy (maximum follow-up of 18 months), with only mild cytokine release syndrome (CRS) and neurotoxicity observed. Long-term efficacy requires validation in larger cohorts (39, 40).

Systemic sclerosis (SSc) is a connective tissue disease characterized by progressive fibrosis of the skin and multiple organ systems (41). Its etiology remains unclear, but accumulating evidence supports a critical role of B cells in the pathophysiology of severe SSc (42, 43). Most patients have detectable autoantibodies (e.g., antinuclear antibodies [ANA], anti-Scl-70 antibodies, and anticentromere antibodies [ACA]), which are valuable for diagnosis and disease activity assessment. To date, 15 cases of SSc treated with CAR-T therapy have been reported (28, 44–48). All enrolled patients had extrapulmonary organ involvement. Following infusion of CD19-targeted CAR-T cells, all patients achieved disease remission with favorable safety profiles: no grade ≥3 CRS or neurotoxicity was observed, nor was long-term B cell aplasia detected. Some patients developed infections and hypogammaglobulinemia. Longer-term remission outcomes require continuous follow-up.

Idiopathic inflammatory myopathies (IIMs) are a heterogeneous group of autoimmune diseases characterized by skeletal muscle inflammation. Based on autoantibody profiles, IIMs are classified into subgroups including dermatomyositis (amyopathic subtype), antisynthetase syndrome, immune-mediated necrotizing myopathy, inclusion body myositis, polymyositis, and overlap myositis (49, 50). B cells play a pivotal role in IIM pathogenesis (51, 52). Histopathological studies have identified B cells and plasmablasts adjacent to T cells in inflamed skeletal muscles. Additionally, rituximab-induced B cell depletion has shown efficacy in some patients with antisynthetase syndrome, further supporting the pathogenic role of B cells (53). Despite current treatments (e.g., glucocorticoids, intravenous immunoglobulin [IVIG], and B cell-targeted agents), antisynthetase syndrome is often refractory, leading to increased mortality (54). To date, 12 IIM patients (presenting with severe myositis or concurrent interstitial pneumonia) have received CAR-T therapy (28, 40, 45, 55–61). All patients exhibited favorable therapeutic responses, with improved symptoms, imaging findings, and laboratory parameters, reduced autoantibody titers, and some achieving first-time autoantibody seronegativity. The therapy had an acceptable safety profile, with no grade ≥3 CRS or high-grade neurotoxicity observed.

Rheumatoid arthritis (RA) is a common chronic systemic autoimmune disease primarily affecting joint synovium, causing pain, swelling, stiffness, and in severe cases, joint deformity and dysfunction (62, 63). Although its pathogenesis is incompletely understood, protein citrullination is a well-recognized trigger of immune responses in RA (64). Serum anti-citrullinated protein antibodies (ACPAs) are specific markers for RA and correlate with disease development and progression (65–67). Thus, B cell depletion to reduce antibody production may be a viable therapeutic strategy (68, 69). Currently, only 4 cases of RA treated with CAR-T therapy have been reported (70, 71), with disease control achieved in all patients. However, only 1 patient achieved 6-month drug-free remission, while the others required maintenance therapy. Therefore, the patient selection criteria and optimal timing for CAR-T therapy in RA remain to be further explored.

### 3.2 Application of bispecific T-cell engager drugs in the treatment of rheumatic autoimmune diseases

Explorations into the use of chimeric antigen receptor T (CAR-T) cells in patients with autoimmune diseases have demonstrated considerable potential; however, they have also revealed several potential risks and limitations, including viral vector integration, prolonged production time, and high costs. Given these factors, alternative approaches for B-cell depletion in the treatment of systemic autoimmune diseases have garnered research interest.

Blinatumomab, the first developed bispecific T-cell engager (BiTE), targets CD19 on B cells and exhibits efficacy even at extremely low concentrations. It enables a single T cell to engage with and sequentially eliminate multiple B cells (72, 73). This agent has demonstrated favorable efficacy in the treatment of acute lymphoblastic leukemia (ALL). Recently, Georg Schett and colleagues conducted the first attempt to administer blinatumomab to patients with multidrug-resistant, refractory rheumatoid arthritis (RA). A total of 6 patients were enrolled in this study (16). All patients had previously received multiple therapies, including conventional disease-modifying antirheumatic drugs (DMARDs) and biologics, yet their disease remained poorly controlled. Following blinatumomab infusion, all patients achieved disease improvement, with no significant adverse events observed during treatment. Neither cytokine release syndrome (CRS) nor neurotoxicity was detected; only three patients developed infections, and one patient experienced mild fever (≤38°C), all of which resolved after symptomatic treatment. During subsequent short-term follow-up, these patients initiated abatacept as adjuvant therapy starting from the 4th month post-treatment, while one patient experienced disease progression prior to abatacept administration. This progression may be associated with dosage, as the dose used in this study was significantly lower than that for hematological malignancy treatment. Subsequently, the same research team enrolled an additional 4 patients (74), diagnosed with RA, Sjögren’s syndrome (SS), idiopathic inflammatory myopathy (IIM), and SS, respectively. All patients had received conventional medications and more than 5 types of biologics previously, but their disease remained active. In this cohort, teclistamab—a bispecific T-cell engager targeting BCMA×CD3—was administered. After treatment, all patients achieved disease control. Following teclistamab infusion, all 4 patients discontinued immunosuppressants; only the RA patient continued glucocorticoid therapy for efficacy maintenance, with a gradual reduction in dosage. Favorable safety profiles were observed in all patients: no neurotoxicity or CRS was detected, and the only adverse events were herpes labialis and upper respiratory tract infections. B cells were rapidly depleted in the short term, and their reappearance was detected at week 12. The newly emerging B cells exhibited phenotypic switching, presenting as naive B cells, with no subsequent disease relapse observed.

Additionally, blinatumomab has been reported for the first time in the treatment of systemic sclerosis (SSc) (75). The patient experienced rapid disease progression within 1 year of diagnosis and had a strong desire to conceive, which restricted the use of many medications. Consequently, the CD19×CD3 bispecific antibody blinatumomab was considered and administered. Following treatment, the patient’s symptoms improved rapidly, with no cytokine release syndrome (CRS), neurotoxicity, infections, or other severe toxic effects observed. During the treatment period, monitoring revealed a significant decrease in B-cell count, which provides further evidence for B-cell depletion therapy in the management of SSc.

Teclistamab is a bispecific T-cell engager (BiTE) targeting B-cell maturation antigen (BCMA) (76). Recently, the first case of a systemic lupus erythematosus (SLE) patient treated with this agent was reported in Germany. The 23-year-old patient had previously received multiple conventional medications and biologics, yet their disease remained active. Following treatment, the patient achieved rapid disease remission: the Systemic Lupus Erythematosus Disease Activity Index 2000 (SLEDAI-2K) score decreased from 20 to 0 at 6 weeks post-treatment, and autoantibody levels also declined rapidly. The treatment was well-tolerated; adverse events included grade 2 cytokine release syndrome (CRS), pneumonia, sinusitis, and hypogammaglobulinemia. No high-grade CRS or neurotoxicity was observed (**Table 2**).

**Table 2:** Published case reports on the use of bispecific T cell engager (BiTE) therapy in rheumatic immune diseases to date (As of December 1, 2025).

Drug	Blinatumomab	Teclistamab	Teclistamab	Blinatumomab
Target	CD19xCD3	BCMAxCD3	BCMAxCD3	CD19xCD3
Location	Germany	Germany	Germany	Germany
Disease	RA	SSc 、SS 、IIM 、RA	SLE	SSc
Patient count	6	4	1	1
Average age (Age range)	50(31~60)	48(24~60)	23	35
Male/Female	3/3	0/4	0/1	0/1
Average disease duration (Duration range)	10.7(3~27)y	123(20~336)m	6y	1y
Disease remission rate	100%	100%	100%	100%
CRS (Grade ≥3)	-	-	-	-
Neurotoxicity	-	-	-	-
Hematotoxicity	-	-	-	+(Thrombocytopenia)
Long-term B-cell dysplasia	NA	NA	-	-
Hypogammaglobulinemia occurrence/non-occurrence	1/5	1/3	1/0	-
Infection occurrence/non-occurrence	3/3	3/1	1/0	0/1
Medication use after treatment		Patients with RA continue steroid use with gradual dosage reduction	Continue oral prednisone until the sixth week after treatment	Medication-free remission

### 3.3 Comparison of CAR-T cells and bispecific T-cell engagers in rheumatic autoimmune diseases

Chimeric antigen receptor T (CAR-T) cell therapy and bispecific T-cell engagers (BiTEs) differ in multiple aspects (**Table 3**), and we will focus on elaborating from the following perspectives:

**Table 3:** CAR-T vs BiTEs.

	CAR-T cell therapy	BiTE
Number and Type of Targets	One, which is the surface antigen of pathogenic cells	Two Components: One End Targets “Pathogenic Cell Surface
		Antigens” (e.g., CD20, BCMA, etc.), and the Other End Binds to T Cell Surface CD3/CD8 Molecules
Administration Route	Single Intravenous Infusion	Multiple Intravenous Infusions/Subcutaneous Injections
*In Vivo* Duration of Action	Relatively Long	Shorter
Efficacy Characteristics	Slow Onset of Action with a Long Duration of Effect	Rapid Onset of Action and Short Duration of Effect
Safety Profile	Higher Rates of Cytokine Release Syndrome (CRS), Neurotoxicity, and Infection	Lower Rates of Cytokine Release Syndrome (CRS), Neurotoxicity, and Infection

#### 3.3.1 Comparison of therapeutic efficacy

Chimeric antigen receptor T (CAR-T) cell therapy is characterized by a relatively slow onset of action, deep remission, and long duration of efficacy (77). Genetically engineered, CAR-T cells can specifically recognize pathogenic cells and independently initiate cytotoxic programs. Moreover, 1–3 weeks after infusion, CAR-T cells undergo rapid proliferation upon recognizing target cells, enabling “bulk elimination” of pathogenic cells (78). Subsequently, a subset of CAR-T cells differentiates into “memory CAR-T cells,” which persist *in vivo* for several months to years, continuously monitoring and eliminating pathogenic cells, thereby achieving long-term remission. However, since CAR-T cell proliferation requires time, a certain period is needed for CAR-T therapy to reach its maximum therapeutic efficacy.

Compared with chimeric antigen receptor T (CAR-T) cell therapy, bispecific T-cell engagers (BiTEs) are characterized by a faster onset of action, weaker depth of remission, and shorter duration of efficacy. Owing to their lack of proliferative capacity, BiTEs start exerting effects within hours after infusion, making them suitable for rapid symptom control during the disease’s acute phase. However, continuous administration is required to maintain therapeutic efficacy, which also gives rise to their limitations of weaker remission depth and shorter efficacy duration.

#### 3.3.2 Comparison of safety profiles

Both bispecific T-cell engagers (BiTEs) and chimeric antigen receptor T (CAR-T) cell therapy can induce cytokine release syndrome (CRS) and central nervous system (CNS) toxicity, but differences exist in the incidence and severity of these adverse events. The pathogenesis of CRS is primarily associated with excessive T-cell activation and the release of large amounts of proinflammatory cytokines (e.g., interleukin-6 [IL-6], tumor necrosis factor-α [TNF-α], interferon-γ [IFN-γ]) (79). Clinical manifestations include fever, hypotension, respiratory distress, and other multisystem symptoms, with severity ranging from mild to life-threatening (77). Following CAR-T cell infusion, the cells proliferate, leading to rapid lysis of a large number of target cells. This process releases excessive cytokines and induces damage-associated molecular patterns (DAMPs), which further stimulate immune cells to secrete additional cytokines—forming a “positive feedback loop” that results in a sharp increase in cytokine concentrations. In contrast to CAR-T therapy, BiTEs do not undergo a proliferation phase; during their mechanism of action, they only bridge a small number of T cells to target cells, without causing excessive target cell lysis in a short period. Consequently, both the incidence and severity of CRS induced by BiTEs are lower than those by CAR-T cells (77). CD3-targeted bispecific T-cell engagers (BiTEs) elicit robust activation across all T-cell subsets, which often results in excessive cytokine secretion and the development of cytokine release syndrome (CRS). To mitigate this adverse effect, CD8-directed BiTE constructs are currently in preclinical (80, 81) and clinical development (NCT06542250), showing promising therapeutic efficacy. While clinical trials evaluating CD8-targeted BiTEs have been actively pursued for oncology indications, there is a paucity of clinical investigations exploring their utility in autoimmune diseases. We therefore look forward to the translational application of CD8-targeted BiTEs in autoimmune disorders. This adverse event is relatively common in the oncology application of these therapies. Currently, tocilizumab, an interleukin-6 (IL-6) receptor antagonist, has become the standard treatment for CRS. Additionally, prophylactic use of corticosteroids and optimization of administration regimens (e.g., stepwise dose escalation) have been shown to reduce the risk of CRS (79). In published case reports of BiTEs and CAR-T cell therapy for rheumatic autoimmune diseases, no high-grade (grade ≥3) CRS has been observed.

Another severe adverse event identified in CAR-T cell therapy for hematological malignancies is immune effector cell-associated neurotoxicity syndrome (ICANS). The pathological mechanism of ICANS has not been fully elucidated; it is currently thought to be associated with blood-brain barrier (BBB) disruption, cerebral vascular endothelial cell activation, and cytokine storm within the central nervous system (CNS). Typical clinical manifestations include language impairment, confusion, seizures, and even cerebral edema, which mostly occur after or overlap with CRS. Fundamentally, CAR-T cells are a type of T cell and possess the ability to actively migrate and penetrate biological barriers. Consequently, a subset of CAR-T cells can cross the BBB. If target antigen-expressing cells (e.g., plasma cells in the CNS) are present within the central nervous system, CAR-T cells will initiate cytotoxic programs, leading to cytokine release and subsequent ICANS. In contrast, BiTEs are inherently large-molecular-weight proteins that rarely cross the BBB to induce ICANS. Therefore, the incidence of ICANS following BiTE therapy is significantly lower than that following CAR-T cell therapy (82).

#### 3.3.3 Comparison of infection risks

Both bispecific T-cell engager (BiTE) therapy and chimeric antigen receptor T (CAR-T) cell therapy increase infection risk due to their induction of immunosuppression, yet differences exist in their infection spectra. Based on infection data from oncology patients previously treated with BiTE or CAR-T therapy: CAR-T therapy requires pre-treatment conditioning chemotherapy (e.g., cyclophosphamide), leading to more pronounced immunosuppression and a higher infection risk. Infections in these patients can be viral, bacterial, or fungal, with a relatively higher incidence of viral infections. In contrast, BiTE therapy requires repeated infusions, and most patients have an indwelling central venous catheter (CVC), making catheter-related bloodstream infections (CRBSIs) the most prominent risk. This is followed by pneumonia and upper respiratory tract infections (83). Currently, few studies have evaluated infection outcomes of BiTE and CAR-T therapies in patients with rheumatic autoimmune diseases. However, data from **Tables 1**, **3** indicate that the incidence of infections associated with these two therapies is comparable in the treatment of rheumatic autoimmune diseases.

## 4 Challenges and future directions for improvement

The complex manufacturing process and exorbitant costs represent significant challenges for chimeric antigen receptor T (CAR-T) cell therapy. Statistics indicate that the current treatment cost of CAR-T cell therapy is as high as several hundred thousand to over one million yuan (20), which imposes an unaffordable financial burden on most patients. Additionally, some patients experience rapid disease progression and cannot wait for the manufacturing period. These factors have collectively limited the widespread clinical application of autologous CAR-T therapy. Consequently, alternative therapeutic strategies, including universal chimeric antigen receptor T-cell therapy (UCAR-T) and CAR-NK cell therapy, have been developed to address these limitations. The T cells utilized in UCAR-T therapy are primarily derived from the peripheral blood of healthy donors (84). Following standardized genetic modification, these cells can be produced in bulk and stored as off-the-shelf products, eliminating the need for patients to undergo a prolonged waiting period for cell manufacturing prior to treatment. In contrast, activated NK cells predominantly secrete IFN-γ and TNF-α, which significantly reduces the risk of severe cytokine release syndrome (CRS). Furthermore, NK cells can be isolated from umbilical cord blood, peripheral blood of healthy donors, or generated via directed differentiation from induced pluripotent stem cells (iPSCs). This enables the scalable production of “off-the-shelf” CAR-NK products without the requirement for patient-specific manufacturing, thereby substantially shortening treatment turnaround time and reducing associated costs (85).

The short half-life of bispecific T-cell engagers (BiTEs) is another pressing issue that requires resolution. This means patients need frequent administration to maintain effective drug concentrations, which increases their treatment burden and inconvenience. To address this problem, researchers are currently exploring various strategies to extend the half-life of BiTEs—such as increasing molecular weight to reduce their metabolic rate—with the goal of improving therapeutic efficacy and patient adherence.

The mortality rate of rheumatic autoimmune diseases is significantly lower than that of malignancies; therefore, treatment safety is a major concern for both patients and clinicians. Adverse effects (e.g., cytokine release syndrome [CRS], neurotoxicity, infections) can occur with both chimeric antigen receptor T (CAR-T) cell therapy and bispecific T-cell engager (BiTE) therapy. The occurrence of these adverse effects not only increases patient suffering but also poses a severe challenge to the safety of clinical treatment, requiring close monitoring and timely management by attending physicians.

Furthermore, both therapies rely on T cells for their therapeutic effects. Chimeric antigen receptor T (CAR-T) cell therapy requires the isolation of a sufficient quantity of T cells for *ex vivo* modification, while bispecific T-cell engagers (BiTEs) function by bridging T cells and target cells. In some patients with autoimmune diseases, T-cell numerical depletion and functional impairment occur due to the disease itself or long-term immunosuppressant use (86), which limits the therapeutic efficacy of both CAR-T and BiTE therapies to a certain extent. In the future, researchers could focus on developing technologies to enhance T-cell function—for instance, using gene editing to upregulate the expression of key receptors on the T-cell surface, thereby boosting T-cell activation and proliferation capacities.

## 5 Future perspectives

### 5.1 Selection of clinical application scenarios

In the application of chimeric antigen receptor T (CAR-T) cell therapy and bispecific T-cell engager (BiTE) therapy, disease activity serves as a key indicator for determining treatment timing and regimens. Clinical studies have shown that CAR-T cell therapy can still induce an objective response rate (ORR) of 60–80% in relapsed/refractory hematological malignancy cases. However, in patients with low tumor burden, it may excessively activate the immune system, leading to a high risk of severe cytokine release syndrome (CRS). Therefore, the NCCN Guidelines recommend CAR-T therapy for patients who have failed ≥3 lines of treatment and have measurable lesions. In contrast, BiTE therapy—characterized by a short half-life and high controllability—is more suitable for maintenance treatment in patients with moderate-to-low disease activity. Thus, based on its data in oncology and the current clinical data of CAR-T therapy in rheumatic autoimmune diseases, CAR-T therapy may be more preferentially used in patients with ultra-refractory rheumatic autoimmune diseases, as well as those with rapidly progressive disease that endangers organ function.

### 5.2 Combination therapy strategies

Chimeric antigen receptor T (CAR-T) cell therapy is characterized by its core advantages of “profound elimination of pathogenic cells and achievement of relatively long-term remission,” while bispecific T-cell engagers (BiTEs) are distinguished by their features of “rapid onset of action and high safety.” For patients with rapidly progressive, refractory severe disease: BiTEs can be used first to rapidly control disease progression. If the disease stabilizes subsequently and the patient still responds to conventional medications or biologics, these conventional agents can be used for maintenance treatment; if there is no response, CAR-T cell therapy can be administered to achieve long-term remission.

## 6 Conclusion

Chimeric antigen receptor T (CAR-T) cell therapy and bispecific T-cell engager (BiTE) therapy represent paradigm-shifting immunotherapeutic modalities that have redefined the therapeutic landscape of rheumatic autoimmune diseases. By harnessing T-cell cytotoxicity to disrupt pathogenic B-T cell crosstalk and selectively eliminate disease-driving cells, these approaches address the unmet clinical needs of patients with relapsed/refractory conditions who fail conventional synthetic disease-modifying antirheumatic drugs (DMARDs) and biologics.

Nevertheless, several critical challenges remain to be addressed. Large-scale, long-term prospective clinical trials are urgently required to validate the safety, efficacy, and durability of remission of both therapies across diverse subtypes of rheumatic autoimmune diseases. Additionally, further investigations are needed to mitigate treatment-related adverse events (e.g., cytokine release syndrome [CRS], infections) and enhance T-cell function in patients with preexisting immune exhaustion. Novel therapeutic targets, optimized delivery systems (e.g., *in vivo* CAR-T, CD8-targeted BiTEs), and personalized treatment algorithms will also be pivotal to improving the clinical utility of these therapies.

In summary, CAR-T and BiTE therapies have opened new avenues for the treatment of rheumatic autoimmune diseases, offering transformative potential for patients with refractory conditions. With the continuous advancement of translational research, clinical validation, and technological refinement, these immunotherapeutic modalities are expected to become integral components of the therapeutic armamentarium, driving significant progress in the field of rheumatic autoimmune disease treatment and improving patient prognosis and quality of life.
